# Global Tendency and Frontiers of Research on Myopia From 1900 to 2020: A Bibliometrics Analysis

**DOI:** 10.3389/fpubh.2022.846601

**Published:** 2022-03-10

**Authors:** Mengyuan Shan, Yi Dong, Jingyi Chen, Qing Su, Yan Wang

**Affiliations:** ^1^School of Medicine, Nankai University, Tianjin, China; ^2^Clinical College of Ophthalmology, Tianjin Medical University, Tianjin, China; ^3^Tianjin Key Laboratory of Ophthalmology and Visual Science, Tianjin Eye Institute, Tianjin Eye Hospital, Nankai University Affiliated Eye Hospital, Tianjin, China

**Keywords:** myopia, public health, bibliometric analysis, global trends, myopia control, refractive surgery, CitNetExplorer

## Abstract

**Background:**

Myopia is one of the most common causes of vision impairment in children and adults and has become a public health priority with its growing prevalence worldwide. This study aims to identify and evaluate the global trends in myopia research of the past century and visualize the frontiers using bibliometric analysis.

**Methods:**

The literature search was conducted on the Web of Science for myopia studies published between 1900 and 2020. Retrieved publications were analyzed in-depth by the annual publication number, prolific countries and institutions, core author and journal, and the number of citations through descriptive statistics. Collaboration networks and keywords burst were visualized by VOSviewer and CiteSpace. Myopia citation network was visualized using CitNetExplorer.

**Results:**

In total, 11,172 publications on myopia were retrieved from 1900 to 2020, with most published by the United States. Saw SM, from the National University of Singapore, contributed the most publications and citations. *Investigative Ophthalmology & Visual Science* was the journal with highest number of citations. *Journal of Cataract and Refractive Surgery* with the maximum number of publications. The top 10 cited papers mainly focused on the epidemiology of myopia. Previous research emphasized myopia-associated experimental animal models, while recent keywords include “SMILE” and “myopia control” with the stronger burst, indicating a shift of concern from etiology to therapy and coincided with the global increment of incidence. Document citation network was clustered into six groups: “prevalence and risk factors of myopia,” “surgical control of myopia,” “pathogenesis of myopia,” “optical interventions of myopia,” “myopia and glaucoma,” and “pathological myopia.”

**Conclusions:**

Bibliometrics analysis in this study could help scholars comprehend global trends of myopia research frontiers better. Hundred years of myopia research were clustered into six groups, among which “prevalence and risk factors of myopia” and “surgical control of myopia” were the largest groups. With the increasing prevalence of myopia, interventions of myopia control are a potential research hotspot and pressing public health issue.

## Introduction

Myopia, also known as short-sightedness or near-sightedness, is one of the most prevalent eye disorders worldwide that lead to vision impairment in young individuals (1). It is one of the five ocular conditions listed as an immediate priority by the World Health Organization's Global Initiative for the Elimination of Avoidable Blindness. A meta-analysis predicted that up to half of the world's population would have myopia by 2050, 10% of which would have high myopia (2). The recent findings around the world imply an increased myopia incidence and myopia progression during the COVID-19 pandemic. The increasing prevalence combined with the rising early onset of myopia, which naturally leads to an increased risk of high myopia (3). High myopia can generate irreversible blindness owing to the secondary changes in the choroid, retina, and sclera (4). Optical interventions, such as spectacles, contact lenses, and refractive surgeries can correct the refractive error; however, they may not prevent high myopia-related complications (5). The large number of patients suffering from myopia and its impact on public health, such as its economic burden and quality of life implications, makes a bibliometric analysis of research studies significant.

Since E.W. Hulme, a British library scientist, first put forward “Statistical Bibliography” in 1922, bibliometric analysis has continued for nearly a 100 years (6). The field started to attract widespread attention with the proliferation of easily accessible online databases and the development of analysis software. Bibliometric analysis is a method that gives a valuable overview of existing academic literature and predicts the development trends of research based on citation reports and content, using mathematical and statistical methods (7). To date, bibliometric analysis has been applied to explore the development and trends of a specific field (8–10).

The research on myopia is so extensive, the number of publications is enormous and the research directions are different which make it difficult to identify the research focus and frontiers in the field. Thus, the study aimed to manifest a general status of global myopia research based on Web of Science (WOS) data from the entire 20th century. The bibliometric method was applied to analyze the research focus, frontiers, and key publications of myopia combined with citation network, and explore the research trend by keywords burst, to provide a comprehensive and promising reference for interested researchers.

## Materials and Methods

### Sources of the Data and Search Strategy

The search for papers to be included in this study was carried out in July 2021 through the Web of Science Core Collection (WOS) provided by Thomson Reuters (Philadelphia, PA, USA). There are many databases available for worldwide research assessment; however, the WOS database is one of the most comprehensive databases with papers dating back to the year 1900 (11). We used the advanced feature and selected the keywords “myopia,” “nearsightedness,” or “shortsightedness” in the title and/or abstracts. The search strategy was as follows: TI = “Myopia” OR AB = “Myopia” OR TI=“nearsightedness” OR AB = “nearsightedness” OR TI = “shortsightedness” OR AB = “shortsightedness.” Only articles and reviews were included as the document types. There were no language restrictions for literature collection. The search covered the period from 1900 to 2020. Data were downloaded from WOS in “plain text” format with “full record and cited references.” The search strategy for the terms related to Myopia was restricted to Title/Abstract to achieve greater accuracy in the results because many reported publications were not related to Myopia if applied to other search fields such as keywords. The use of title/abstract search is recommended in the bibliometric studies in contrast to the title-abstract-keywords search query because it substantially increases the specificity with minimum loss of sensitivity.

### Bibliometric Software

In bibliometric analysis, the annual number of publications, prolific countries and institutions, core author and journal, paper citations, keywords, and bibliometric indicators are presented through descriptive analysis. The built-in analysis tool of Web of Science can create the citation network, but it is limited to offering the connections that exist between the citations of specific groups of articles and the co-authorship between the specific items. We applied bibliometric software to this study due to this reason. CitNetExplorer software was used to evaluate the development of scientific research within a specific field, which enable the researcher to visualize the citation networks and the relationship among these articles (12). VOSviewer software offers text mining functionality that can be used to construct and visualize co-occurrence networks of important terms extracted from a body of scientific literature, represented as nodes and links (13). The nodes size represented the number, and the links between the nodes reflected the partnership between the items. The graphic display ability of CiteSpace is not as strong as that of the VOSviewer, but it has the unique burst analysis function of keywords, which can demonstrate the changes in the hot spots in this field (14).

### Data Analysis

The data from the WOS database was imported to the bibliometric software to produce visualization results and quantitative analysis for researchers. For this analysis, the most common bibliometric indicators were used: the number of publications, the number of citations. Microsoft Excel was used to arrange and sort the data, and extract the top results. The publication citation network was calculated using CitNetExplorer software. The setting of the clustering parameters resolution was set at 1.20 and a minimum cluster size of 1,000 articles. The co-authorship networks of countries, authors, organizations were made by VOSviewer, respectively. We chose the optimized parameter, which is described in detail in each figure notes. The burst keywords were assessed using CiteSpace software with the following parameters: time slicing (1990–2020), years per slice (1), term source (title, abstract, author keyword, keyword plus), node type (keyword), selection criteria (top 30).

## Results

### Description of Publication

#### Growth Trends of Publications

**Figure 1:**
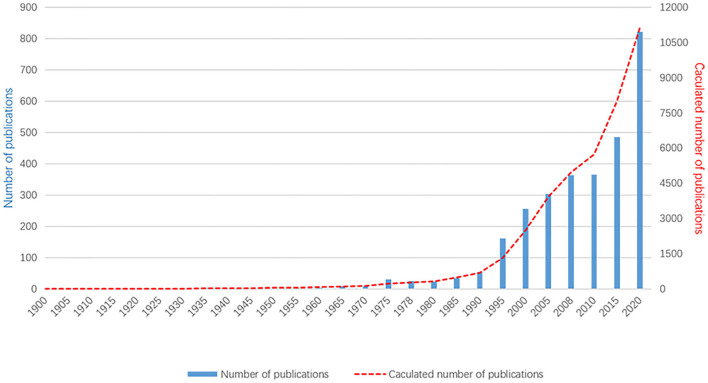
The annual number of published myopia studies, 1900–2020. As the data in 2021 are still updated, they are not included in this figure.

Based on the WOS database analysis, 11 172 documents on myopia published between 1900 and 2020 were retrieved. The first article on myopia was published in 1907. Prior to 1990, this field of research had not received much attention. Since 1991, the number of articles published increased gradually from 100 publications to over 400 after 2011 (Figure 1). There were 822 articles published in 2020. In 2021, 429 articles have been published as of June, and the number is likely to increase.

#### Distribution of Countries

According to the retrieved articles, the articles on myopia originated from 127 countries. Table 1 shows that the United States accounted for the most number of articles published (19.82%), followed by China and Australia. Studies from the United States were cited 105 738 times, ranking first among all countries, followed by Australia and China. The collaboration relationship was analyzed using VOSviewer. As shown in Supplementary Figure 1, the United States (USA), the largest node, is the most active country in this field. The cooperation map showed that the USA intensively collaborated with many countries in myopia fields, such as Germany, France, and Spain.

**Table 1:** Top 10 most influential countries in myopia research.

**Rank**	**Country**	**Number of citations**	**Number of publications**
1	USA	105,738	2,786
2	Australia	35,433	913
3	China	34,961	2,088
4	England	26,674	764
5	Germany	22,381	839
6	Japan	20,944	664
7	Singapore	20,569	433
8	Spain	13,484	508
9	Italy	9,452	416
10	Canada	8,518	293

#### Distribution of Authors

According to the retrieved results, over 71,292 authors contributed to myopia research. Table 2 lists the 10 most productive authors in the field of myopia research. Among all authors, Saw SM contributed the most publications (175), the most citations (10,448 times). As shown in Supplementary Figure 2, the cooperative relationships among the productive authors are close, except for the group marked in yellow. There are several co-authorship groups, such as the red group with Saw SM as the core, the green group with Smith EL as the core, and the blue group with Mutti DO as the core.

**Table 2:** Top 10 most influential authors for myopia studies.

**Rank**	**Author**	**Number of citations**	**Number of publications**
1	Saw SM	10,448	175
2	Mitchell P	8,384	121
3	Wong TY	7,161	107
4	Ohno-Matsui K	5,779	140
5	Wallman J	4,834	27
6	Mutti DO	4,736	68
7	Zadnik k	4,665	58
8	Morgan IG	4,146	50
9	Jonas JB	4,033	108
10	Schaeffel F	3,849	104

#### Distribution of Journals

Based on the retrieved results, the articles on myopia research were distributed among 164 journals. The top 10 journals that published articles on this topic are listed in Table 3. According to the citations, *Investigative Ophthalmology & Visual Science* and *Ophthalmology* ranked first and second, respectively. The *Journal of Cataract and Refractive Surgery* published the largest number of myopia articles (834 papers), followed by *Investigative Ophthalmology & Visual Science*. Among the top 10 journals, eight were from the USA, one was from the United Kingdom, and one from Germany.

**Table 3:** Top 10 influential source journals for myopia studies.

**Journal**	**Country**	**Number of citations**	**Number** **of publications**
Investigative Ophthalmology & Visual Science	USA	37,443	708
Ophthalmology	USA	37,220	491
Journal of Cataract and Refractive Surgery	USA	23,368	834
American Journal of Ophthalmology	USA	17,993	414
Journal of Refractive Surgery	USA	16,539	701
Optometry and Vision Science	USA	12,554	437
British Journal of Ophthalmology	UK	10,886	313
Archives of Ophthalmology	USA	10,062	157
Vision Research	UK	8,180	155
Ophthalmic and Physiological Optics	USA	6,621	260

#### Distribution of Organizations

As shown in Table 4, the top 10 organizations published 2,161 articles. Citation analysis showed that the National University of Singapore had 14,968 citations and ranked first. According to the publications, National University of Singapore and Sun Yat-sen University ranked first with 285 publications. The University of Melbourne, with 264 articles, ranked third. In the knowledge domain map of collaboration among main research organizations, 45 countries, 6 clusters, and 874 links were displayed and selected. As shown in Supplementary Figure 3, the National University of Singapore has the highest number (35 links) and the strongest link strength (629).

**Table 4:** Top 10 influential organizations for myopia studies.

**Rank**	**Organization**	**Country**	**Number of citations**	**Number of publications**
1	National University of Singapore	Singapore	14,968	285
2	University of Sydney	Australia	10,709	179
3	University of Melbourne	Germany	10,592	264
4	Singapore National Eye Center	Singapore	10,342	223
5	Singapore Eye Research Institute	Singapore	9,641	192
6	Sun Yat-sen University	China	6,381	285
7	Tokyo Medical & Dental University	Japan	6,201	160
8	Hong Kong Polytechnic University	China	4,311	158
9	Capital Medical University	China	2,980	176
10	Fudan University	China	2,836	239

#### Top Cited Publications

The top 10 cited references are summarized in Table 5. The top 10 papers were co-cited over 6,000 times in total, and the first was co-cited more than 800 times, while the 10th was cited 516 times. Additionally, the fifth paper was the only one published before the year 2000 cited 538 times. The top 10 cited references mainly focused on the prevalence and risk factors of myopia, which is consistent with the latest burst keyword.

**Table 5:** Top 10 cited papers in myopia citation network.

**Ranking**	**Title**	**Author**	**Year**	**Number of citations**
1	Global prevalence of myopia and high myopia and temporal trends from 2000 through 2050	Holden BA	2016	844
2	The multifunctional choroid	Nickla NL	2010	773
3	Myopia	Morgan IG	2012	728
4	Outdoor activity reduces the prevalence of myopia in children	Rose KA	2008	602
5	The relationship between glaucoma and myopia—the blue mountains eye study	Mitchell P	1999	538
6	Homeostasis of eye growth and the question of myopia	Wallman J	2004	586
7	Enhanced depth imaging optical coherence tomography of the choroid in highly myopic eyes	Fujiwara T	2009	537
8	Prevalence and risk factors for refractive errors in adult Chinese in Singapore	Wong TY	2000	535
9	Myopia and associated pathological complications	Saw, SM	2005	533
10	Refractive error and visual impairment in urban children in southern China	He MG	2004	516

### Myopia Research Keywords and Tendency

**Figure 2:**
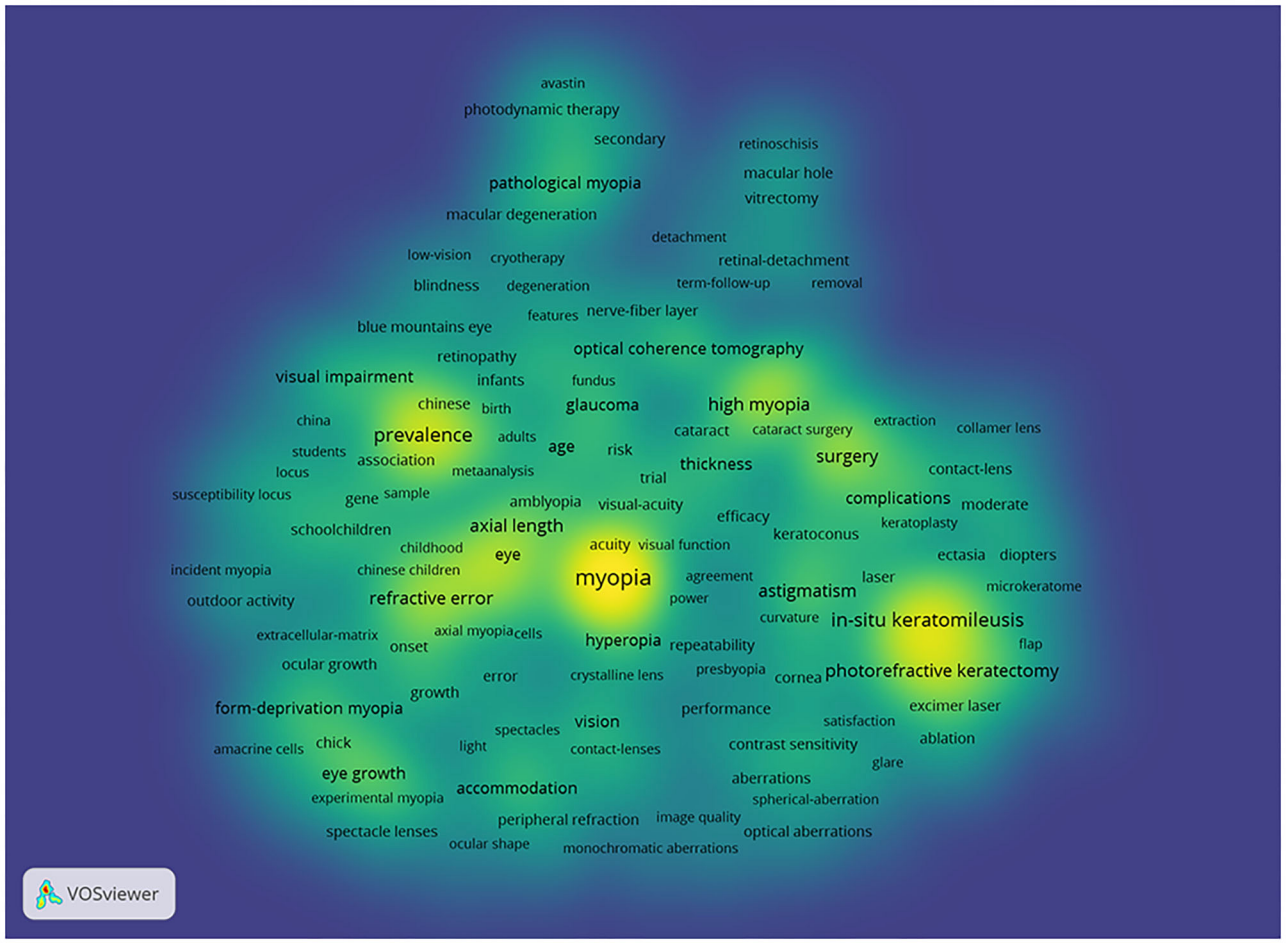
Density visualization for keywords in co-occurrence network map. Each keyword in the density visualization has colors that indicates its appearing frequency. Keywords in yellow emerge more frequently, while green emerge blue less frequently.

**Figure 3:**
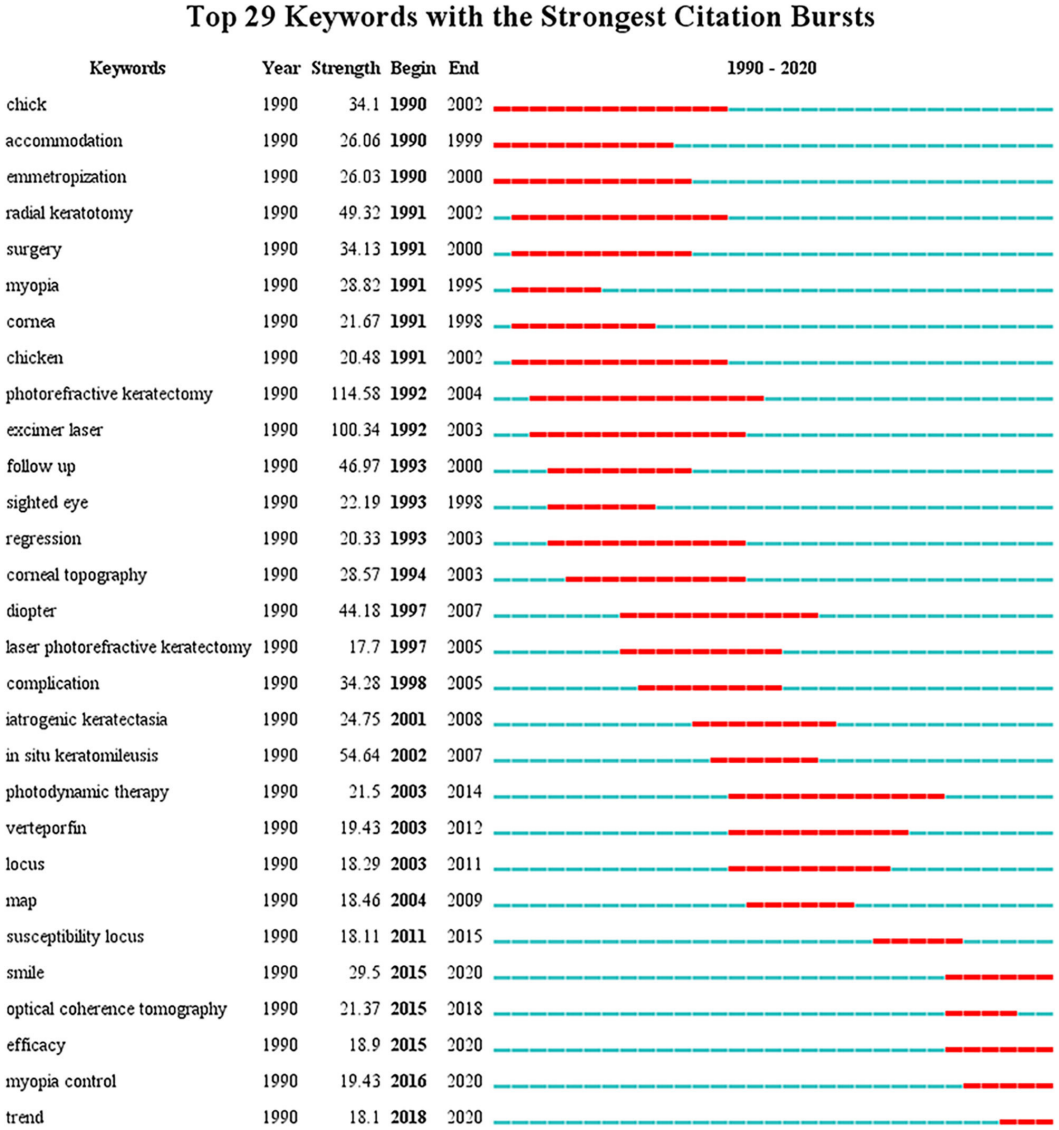
The top 29 keywords with the strongest citation bursts in myopia research from 1990 to 2020. The blue lines represent the base timeline, while the red segments represent the burst duration of the keywords.

Through co-occurrence analysis, the keywords were visualized by density network map (Figure 2). The keyword “*in-situ* keratomileusis,” “prevalence,” and “photorefractive keratectomy” turned out to be significant. These keywords were the core keywords in myopia research. The top 29 keywords with the strongest citation bursts were extracted via keyword burst analysis from 1990 to 2020 (Figure 3). “Chick,” the first keyword detected, appeared in 1990 and lasted for 12 years. Among the 29 keywords, “photorefractive keratectomy” had the highest burst strength (114.58) in the steady development stage. The latest keywords in the rapid development stage were “myopia control” and “trend.”

### Myopia Research Citation Network

**Figure 4:**
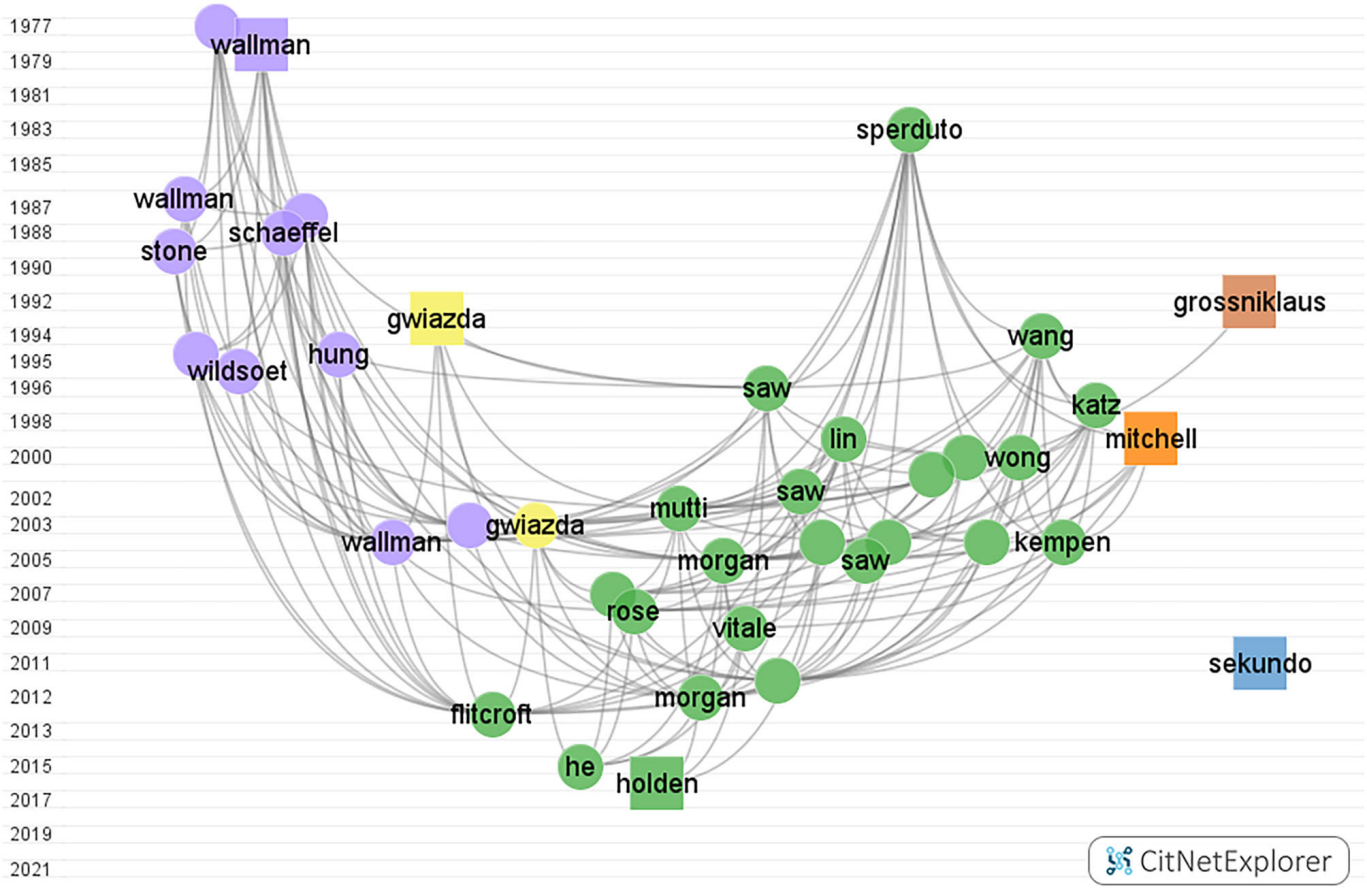
Myopia citation network graph from CitNetExplorer from 1900 to 2000. The vertical axis coordinates indicate the publication year. Each dot/square indicates a publication which is labeled with the last name of the first author. Each color marks a group. Group 1 to group 6, in turn, were colored green, blue, purple, yellow, orange, and brown, respectively. The square represents the publication with the highest citation score in each group.

Figure 4 shows the main publication citation network of myopia. Based on the clustering function, each publication would be assigned to six research focuses. Each color marks a group. Each direction has its own citation network, which consists of publications that are strongly linked to each other.

The color green represents the prevalence and risk factors of myopia group, containing 2,711 publications, and almost 32% of the total citation score. The color blue represents the surgical control of myopia group with 3,059 publications, and the total citation score was 34,557. The color purple represents the pathogenesis of myopia group, where 1,456 articles were found within the network. The color yellow represents the optical interventions of myopia group. The color orange represents the myopia and glaucoma group. The color brown represents the pathological myopia group.

Supplementary Figures 4–9 show the citation network of each of the six research focuses.

## Discussion

Bibliometric analysis is one of the most prominent methods for researchers to identify and predict new trends in potential topics. Moreover, it has been widely recognized as an alternative tool for evaluating academically detailed information in the library and information science. There has been some studies on myopia, but their coverages were limited in a single area of myopia research and did not include keywords bursts in its analysis (15, 16). In this study, we conducted a comprehensive bibliometric analysis of the literature available on myopia from 1900 to 2020; six groups were identified within the citation network, and keywords bursts detection was performed.

### Global Contribution in Myopia Research

Trend variations in publication quantity can reflect changes in knowledge on a certain subject. The number of documents on myopia studies has been through three stages: the initial stage (before 1991), steady development stage (from 1991 to 2011), and rapid development stage (after 2011). In the initial stage, the total publications were about 700, the annual average amount was about 7 papers. The increase in the global pattern of published papers was particularly prominent after the 1990's, which may be associated with a shift in focus toward newly developed techniques for refractive surgery with better safety and effectiveness (17, 18).

International cooperation has become one of the main scientific research patterns among countries. In the current study, the United States was found to be the leading country in myopia research, accounting for 19.82% of total publications and the highest number of citations. According to the connection between various nodes, the United States attaches great importance to exchanges and cooperation in the academic community. This also explains why the United States has greater output to some extent. It can be speculated that adequate funding, advanced techniques, and equipment are essential factors. However, it is equally important that numerous authors from the United States produced high-quality research with good communication and collaboration with others. Smith EL of the University of Houston conducted animal experiments on myopia and explored the role of visual signals on refractive development. He stated that optical defocus can regulate eye growth and myopia progression by a small but statistically significant amount (19). In the initial stage, Curtin BJ was the most cited author, who was from the USA as well. The most cited article found that high myopia was associated with abnormal proteoglycans in sclera which changed the size and organization of collagen fibrils (20). In terms of the authors' analysis, Saw SM from Singapore was the most cited author in the steady development stage and the rapid development stage. In 2009, the article titled “Outdoor activity and myopia in Singapore teenage children” was published in the British Journal of Ophthalmology. This study suggested that outdoor activity may protect against the progression of myopia in children (21). Top source journals also came from the United States, with the *Journal of Cataract and Refractive Surgery* (JCRS) being the most prolific in publishing myopia research. Synthetically, *Investigative Ophthalmology & Visual Science* was the most influential journals, which ranked first of citations. As for the research institutions, among the top 10 institutions, eight institutions were located in Asia, which was in accordance with the increasing prevalence of myopia in East and Southeast Asia (22, 23).

### Focus in Myopia Research

Research focus represents the combination of clinical subjects and basic research and indicates the increasing or emerging themes in the field of myopia. In bibliometrics, the cluster function showed that all publications can be separated into six groups, and each group was summarized to a specific theme. With reference to the characteristics and status of myopia research, the following six groups are discussed.

#### Prevalence and Risk Factors of Myopia

The sharp rise in the myopic population increased its socioeconomic burden and posed a public health problem worldwide. Thus, the prevalence of myopia and its risk factors have gained widespread attention (Supplementary Figure 1, Green). Carrying out scientific epidemiological research on myopia is a mainstay for exploring related influencing factors for myopia, which are critical for intervening on its onset and progression. Before 1980, little was known about the distribution of myopia in the worldwide population. East and Southeast Asia showed the highest prevalence, reaching 80–90% at 18 years of age, which was much higher than that of Central Europe and Central Asia (24, 25). A meta-analysis has suggested that by the year 2050, nearly 50% of the world's population will have myopia, and approximately 10% will be high myopic. This is the most cited paper in this myopia area published by Holden et al. in 2016 (2). The first prospective study of the risk factors for myopia, showed that earlier age of onset of myopia was a risk factor for the development of high myopia, which induced non-correctable visual impairment or blindness (3). The genetic pool has changed little over the past few decades, but the changes in the environmental factors may be responsible for the rapid increase in the prevalence of myopia (26). It seems that school myopia is multifactorial, strongly associated with intensive educational pressure and limited outdoor activities (27). In terms of educational level, there was a high prevalence of myopia in boys attending Orthodox schools in Israel compared with their peers attending secular schools (27). The mechanism involved is unclear; however, near-work requires more accommodation which may stimulate eye growth. According to the prevailing view, longer time spent outdoors can prevent myopia (28). The available data revealed that high-intensity outdoor light may act as a protective factor by adding retinal dopamine concentrations and thus preventing myopia (29).

#### Surgical Control of Myopia

With the development of laser technology in modern ophthalmic surgery, refractive surgery is an important research area to improve the refractive status (30) (Supplementary Figure 5, Blue). Refractive surgery, a safe and effective measure that corrects refractive errors, is generally not recommended until refractive development has stabilized around the age of eighteen. The first case of radial keratotomy surgery was reported by Fyodorov in 1979; however, it has been replaced by cornea laser surgery owing to its lower security and poor efficiency (31). The keratorefractive surgery revolution began with surface ablation techniques. Surface ablation is potentially suitable for high myopia and thin corneas due to the relatively thicker residual stromal thickness (32). Nonetheless, corneal haze and myopic regression may be more common after surface ablation (33). Laser-assisted *in situ* keratomileusis (LASIK) is a new revolution, which has been the standard refractive surgery used for treating myopia since the 1990's (34). A review of LASIK outcomes was published in 2016 by Sandoval et al. (35). The authors reported that the spherical equivalent refraction (SE) and the uncorrected distance visual acuity of eyes both obtained pretty good correction effects and up to 98.8% of patients were satisfied with their outcome. However, complications associated with this procedure are not rare, such as free cap, buttonhole flap (36, 37). Presently, small-incision lenticule extraction (SMILE) has emerged as a novel surgery for myopia with the introduction of the femtosecond laser platform. The most frequently cited article in this cluster was published in 2011 and written by Sekundo et al. (38). The authors acknowledged that SMILE was a promising new minimally invasive procedure. SMILE has shown a reduced degree of dry eye symptoms and higher-order aberrations relative to LASIK (39, 40). These advantages may stem from small side cut and lower laser energy which retains corneal nerve and reduce inflammatory responses to a large extent (41, 42). Refractive surgery has already achieved excellent visual outcomes; however, the next challenge for clinicians is to choose the best refractive surgery method for each patient. Recently, with the addition of artificial intelligence in preoperative evaluation, data derived from corneal topography, biometry, and aberrometry can optimize customized refractive surgical strategies (43, 44). Based on the keywords, myopia correction remains centered on the corneal refractive operation. Intraocular surgery, which avoids the risk of corneal ectasia, is also developing rapidly (45).

#### Pathogenesis of Myopia

At present, one of the areas that require further studies is the pathogenesis of myopia (Supplementary Figure 6, Violet). The mechanisms underlying axial elongation may provide scientific clues for the prevention and control of this global epidemic. From birth to adolescence, the eye achieves a close match between the power of its optics and its axial length, with images that are focused on the retina without accommodative effort (emmetropia) (46). Interestingly, Rucker FJ discovered that the retina may be using contrast perception to decode the sign of defocus using contrast perception. The chromatic signals of longitudinal chromatic aberration may rely on cone modulation to provide a direction signal for accommodation and eye growth (47, 48). Within the past several decades, it has become clear that alterations in the visual experience can provoke myopia in animal models (19, 49). For instance, form deprivation causes axial myopia through reduced visual stimulation, which is different from defocused-myopia related to the central system (50). The most frequently cited publication is the paper written by Wallman et al.. The authors reported that reading for long periods may disturb the homeostasis in the posterior globe, resulting in scleral remolding (51). The advances in clinical and basic experiments are mainly in the posterior ocular segment (52–54). Recently, Zhou et al. proposed a hypothesis that scleral hypoxia is a target for myopia control in 2018 (54). They speculated that special visual stimulation regulates the choroidal blood, thus initiating scleral hypoxia, leading to the onset and progression of myopia and axial elongation. Therefore, choroidal blood perfusion might be a “rapid predictive index” for myopia management (55). However, despite the progress, the chain of events of choroidal signals and scleral targets are still largely unknown. These clues may direct researchers to improve the understanding of myopia through the expansion of omics and big data analysis. In addition, some studies showed that atropine was found to increase the release of dopamine, strengthen the sclera, and increase the choroidal blood (56, 57).

#### Optical Interventions of Myopia

As for group 4, the frequently cited articles were related to the optical interventions study (Supplementary Figure 7, Yellow). This group was led by the cross-sectional study by Millodot et al., published in 1981, in which the effect was measured between peripheral refraction and ametropia, where peripheral hyperopic defocus accelerated the onset of myopia (58). The first optical intervention was based on the reasoning that there was a relationship between myopia and excessive accommodation. The use of single-vision lenses (SVLs) was the mainstream method to correct myopia; however, SVLs poorly controlled myopia progression. It is not possible to clear the causality between peripheral retinal defocus and central myopia. However, it has been widely recognized that peripheral hyperopia can promote myopia progression (19, 59). Thus, peripheral defocus spectacle lenses (PDSL) and orthokeratology that were precisely designed to reduce peripheral retinal defocus, were effective (60, 61). The most cited publication was the article by Jane Gwiazda et al., which was published in 2003 in Investigative Ophthalmology & Visual Science (62). The authors reported that compared with SVLs, PALs limited the progression of myopia during the 1st year. The evaluation of visual quality in the human eye has always been an important issue in the field of ophthalmology and visual optics, which generally focus on the correlation analysis of visual acuity, wavefront aberrations, and contrast sensitivity (63–65). Previous research has indicated that visual quality was negatively related to the degree of myopia. It decreases gradually and concomitantly as the degree of myopia increases, while contrast sensitivity decreases and higher-order aberrations increase (66, 67).

#### Myopia and Glaucoma

The connection between myopia and glaucoma clinically has also been a significant research topic in recent years (Supplementary Figure 8, Orange). Glaucoma is the leading cause of irreversible blindness worldwide, and primary open-angle glaucoma (POAG) is the major type of glaucoma. Glaucoma is strongly linked with the onset and progression of myopia, with homogeneity in structural and functional changes (68). The most cited paper was published in 1999 in Ophthalmology by Mitchell et al. and was ranked fifth in the top 10 cited publications (69). The study revealed that myopia, an independent risk, increased the prevalence of glaucoma by 2 or 3-fold. A study from Korea found a positive trend between increased myopic refractive error and POAG prevalence (70). Recently, a meta-analysis by Ahnul Ha et al. corroborated this finding: every diopter in myopia increases the risk of glaucoma by ~20% (71). Thus, high myopia is now regarded as a risk factor for glaucoma (72–74). With increased axial length, high myopia appears to have optic disc morphological changes and optic nerve fiber layer defects, accelerating visual field defects (72, 73). In this group, the newest, most cited citation centers on myopia-related optic disc changes. Saw SM et al. reported that tilted discs and peripapillary atrophy were common in Singaporean adolescent children, which were similar to the pathological changes in glaucoma (75). Retinal degeneration makes it difficult to detect glaucoma with severe myopia, which requires a myopic normative database for analysis (76). Taken together, there is a need for a multimodal approach combining structural images with functional assessments to overcome the clinical diagnostic dilemmas of myopic eyes with glaucoma (77).

#### Pathological Changes of Myopia

Pathological myopia has gained attention because of its sustained axial elongation and irreversible fundus degeneration that leads to severe vision loss (Supplementary Figure 9, Brown). This cluster focuses on myopic maculopathy, especially in myopic choroidal neovascularization (mCNV) (78). The reasons behind the development of myopic maculopathy are not clear, but evidence has shown that excessive axial elongation weakens the retina, choroid, and sclera, which is accompanied by vascular complications and degeneration (79, 80). Curtin and associates first proposed five fundus changes in myopia associated with axial elongation in 1970 (81). This classification did not cover all myopic maculopathy lesions. The development of fundus imaging technology facilitated a clearer visualization of myopic maculopathy. Grossniklaus et al. published an article in 1992 that described the pathological changes in pathological myopia and was the most cited paper in this group (82). A simplified classification system was proposed by Ohno-Matsui et al. (83). In this system, myopic maculopathy lesions were classified into five categories: no myopic retinal lesions (category 0), tessellated fundus only (category 1), diffuse chorioretinal atrophy (category 2), patchy chorioretinal atrophy (category 3), and macular atrophy (category 4), in combination with the three “plus signs” of lacquer crack, myopic choroidal neovascularization, and the Fuchs spot. Choroidal neovascularization may develop in 5–10% of individuals with pathological myopia (84), which is easily diagnosed using optical coherence tomography, optical coherence tomography angiography, and fundus fluorescein angiography. Photodynamic therapy with verteporfin (vPDT) was the first approved treatment for mCNV. Nevertheless, vPDT also resulted in chorioretinal atrophy and influenced the final visual outcome (85, 86). In recent years, intravitreal anti-endothelial growth factor (anti-VEGF) injection has become the first-line treatment for CNV secondary to pathological myopia (87). Anti-VEGF agents, such as ranibizumab and aflibercept, can control neovascularization and reduce macular edema, thereby improving visual acuity (88). However, patients do not acquire significant benefits in long-term vision with this treatment due to the development of mCNV-related macular atrophy (89).

### Tendency in Myopia Research

The strongest citation burst keywords were considered the indicators of research trends in basic and clinical research. As a result of the fewer numbers of annual average publications, no distinct research trend was observed in the initial stage. Despite this, the most cited paper showed that lid fusion led to elongation of the eye globe and varying degrees of myopia in monkeys published in 1977 (90).

In the steady development stage and rapid development stage, we conducted the keywords burst to explore myopia tendency and frontiers. According to the keyword co-occurrence chronology, the most prominent keywords in steady development stage are “photorefractive keratectomy,” “excimer laser,” and “*in situ* keratomileusis,” indicating that the study focus was refractive surgery. Experts such as Da Vinci proposed the first theories as to the source of refractive errors long ago. At the Aerospace Medical Association, an intervention reported excimer laser can be used to change the corneal shape, piqued experts' curiosity (91). McDonald and colleagues became the first to utilize an excimer laser in the human eye with myopia in 1988 (92). As the number of surgeries rose, the drawbacks of photorefractive keratectomy, started to emerge such as postoperative pain and corneal haze. In the 1990's, Pallikaris with colleagues proposed a novel surgical procedure (LASIK) that merged the microkeratome with the excimer laser, and it has now become a wildly used refractive technique (93). In addition to clinical studies, animal studies had a high profile within the steady development stage. Notably, the keyword chick appeared twice in the analysis. Several animal studies have demonstrated environmental factors can exert a significant effect on myopia. In this regard, we found that Wildsoet CF is the most cited author at the second period. Most attention was drawn to an article published in 1994 showing that hyperopic defocus induced with negative lens led to increased ocular length and choroidal chinning, whereas myopic defocus induced with positive lens led to decreased ocular elongation and choroidal thickening (94). According to recent studies by Wildsoet CF, the Bone Morphogenetic Proteins (BMP 2, 4, and 7) gene is down-regulated with form-deprivation and hyperopic defocus, and up-regulated with myopic defocus in chicks, which exhibited regional differences in retinal pigment epithelium. Consequently, it is tempting to speculate that BMPs played a crucial role in ocular growth signaling (95, 96).

During the rapid development stage, we have observed that a new chapter in refractive surgery was opened with the application of the femtosecond laser in ophthalmology. SMILE has been the most recent, strongest burst, which has been approved by the Food and Drug Administration for the treatment of myopia and astigmatism preventing iatrogenic dry eye and allowed better spherical aberration control (97, 98). Beyond 2020, refractive surgery might be guided by artificial intelligence to make precise decisions regarding surgery details and improve the quality of the retinal image (99, 100). In the third phase, the keywords “myopia control” and “trend” show that there is an urgent need for society to take interventions on myopia, which are the new research hotspots in this field. With the rapidly growing prevalence of myopia already at epidemic levels in some regions and imposing a heavy public health burden (5), the scientific interest in myopia control is growing with each passing day. The amount of scientific papers has increased excessively concerning myopia control. Myopia management strategies consist of two parts: the prevention of myopia onset and slowing the progression of myopia (99). The current control measures, including optical, pharmacological, behavioral, and surgical interventions. On the basis of a series of influential studies, increased time outdoors could preclude high engagement in near-work activities and exposure to ultraviolet radiation, which is more meaningful in preventing myopia (21). A recent review suggested that the changes in SE and axial length were better in the outdoor group than that in the control group (101). Studies using animal models reported that bright light may play an inhibitory role in response to imposed form-deprivation (102). When compared with other measures, wearing optical devices is a convenient method that reduces the peripheral hyperopic defocus to limit myopic progression. Substantial evidence from animal research indicates that hyperopic defocus induces axial elongation whereas myopic defocus inhibits the growth of axial elongation (103). For the present research outcomes, pharmacological treatment was insufficient to cluster into groups in the myopia field. Atropine is the most extensively studied medicine in slowing progression. Low-dose atropine (0.01%) seemed to be the most effective treatment and had a lower risk of rebound according to the Atropine for Treatment of Myopia (ATOM2) study (104). It was suggested that atropine may exert its function by altering choroidal thickness to reduce scleral proteoglycan synthesis (105, 106). As for surgical interventions for the control of myopia, scleral reinforcement to slow ocular elongation has a long history. The revitalized interest arose from collagen cross-linking scleral strengthening (CCL) controlling scleral biochemistry which has involved animals only (107). Recently, Bullimore et al. reported that the potential benefits of myopia control outweigh the risks (108). Moreover, applying artificial intelligence to ocular data may provide a better approach for reducing public burden focusing on myopia control. Generally, the preventive strategies aim to avoid younger age of myopia onset or lower the risk of high myopia; therefore, the sooner the intervention, the better is the outcome and the impact on public health. In short, the efforts of myopia controls could have a profound impact on public health.

### Strengths and Limitations

The present study is the first bibliometric analysis of myopia performed using the literature from the entire 20th century. To acquire deep insight into myopia research, VOSviewer was used to identify the hotspots and major clusters in this field. However, despite these advantages, several limitations should be noted in our study. The data were only retrieved from the WOS database and did not include other medical databases such as PubMed and Scopus. As reported, the WOS database has more accuracy in document type assignment than Scopus (109). The WOS was preferred over PubMed due to a unique citation report function (11). Regardless, the WOS database is the most commonly used reference database for bibliometric analysis.

## Conclusions

Based on the bibliometric analysis, myopia has been growing as a core research area. United States has the most significant academic impact on myopia studies. The most productive and cited institution was the National University of Singapore. Saw SM is one of the key researchers in this field. The priority themes involved the prevalence and risk factors of myopia and surgical control of myopia. With the increasing prevalence of myopia, the interventions of myopia control are the potential research hotspot and pressing issue. Taken together, these analysis results should help researchers realize the current state and provide promising directions for future research.

## Data Availability Statement

The datasets presented in this study can be found in online repositories. The names of the repository/repositories and accession number(s) can be found in the article/Supplementary Material.

## Author Contributions

MS and YD did this bibliometrics analysis and drafted manuscript. MS and JC organized the manuscript writing. QS searched strategy. YW reviewed the manuscript. All authors contributed to the article and approved the submitted version.

## Funding

This research was funded by the National Natural Science Foundation of China, Grant No: 81873684.

## Conflict of Interest

The authors declare that the research was conducted in the absence of any commercial or financial relationships that could be construed as a potential conflict of interest.

## Publisher's Note

All claims expressed in this article are solely those of the authors and do not necessarily represent those of their affiliated organizations, or those of the publisher, the editors and the reviewers. Any product that may be evaluated in this article, or claim that may be made by its manufacturer, is not guaranteed or endorsed by the publisher.
