# Corrigendum: Global tendency and frontiers of research on myopia from 1900 to 2020: A bibliometrics analysis

**DOI:** 10.3389/fpubh.2022.1063615

**Published:** 2022-10-26

**Authors:** Mengyuan Shan, Yi Dong, Jingyi Chen, Qing Su, Yan Wang

**Affiliations:** ^1^School of Medicine, Nankai University, Tianjin, China; ^2^Clinical College of Ophthalmology, Tianjin Medical University, Tianjin, China; ^3^Tianjin Key Laboratory of Ophthalmology and Visual Science, Tianjin Eye Institute, Tianjin Eye Hospital, Nankai University Affiliated Eye Hospital, Tianjin, China

**Keywords:** myopia, public health, bibliometric analysis, global trends, myopia control, refractive surgery, CitNetExplorer

In the published article, an author name was incorrectly written as “Yan Wan”. The correct spelling is “Yan Wang”.

The authors apologize for this error and state that this does not change the scientific conclusions of the article in any way. The original article has been updated.

## Publisher's note

All claims expressed in this article are solely those of the authors and do not necessarily represent those of their affiliated organizations, or those of the publisher, the editors and the reviewers. Any product that may be evaluated in this article, or claim that may be made by its manufacturer, is not guaranteed or endorsed by the publisher.

