# Supplementary material for: Global Tendency and Frontiers of Research on Myopia From 1900 to 2020: A Bibliometrics Analysis
Source: Front Public Health. 2022 Mar 10;10:846601. doi: 10.3389/fpubh.2022.846601 (PMC8960427; doi:10.3389/fpubh.2022.846601)
Supplement: Supplementary file 1 [file Data_Sheet_1.PDF]

frontiers

Supplementary Material

1 Supplementary Data

Supplementary Material should be uploaded separately on submission. Please include any supplementary data, figures and/or tables. All supplementary files are deposited to FigShare for permanent storage and receive a DOI.

Supplementary material is not typeset so please ensure that all information is clearly presented, the appropriate caption is included in the file and not in the manuscript, and that the style conforms to the rest of the article. To avoid discrepancies between the published article and the supplementary material, please do not add the title, author list, affiliations or correspondence in the supplementary files.

2 Supplementary Figures and Tables

For more information on Supplementary Material and for details on the different file types accepted, please see here. Figures, tables, and images will be published under a Creative Commons CC-BY licence and permission must be obtained for use of copyrighted material from other sources (including re-published/adapted/modified/partial figures and images from the internet). It is the responsibility of the authors to acquire the licenses, to follow any citation instructions requested by third-party rights holders, and cover any supplementary charges.

2.1 Supplementary Figures

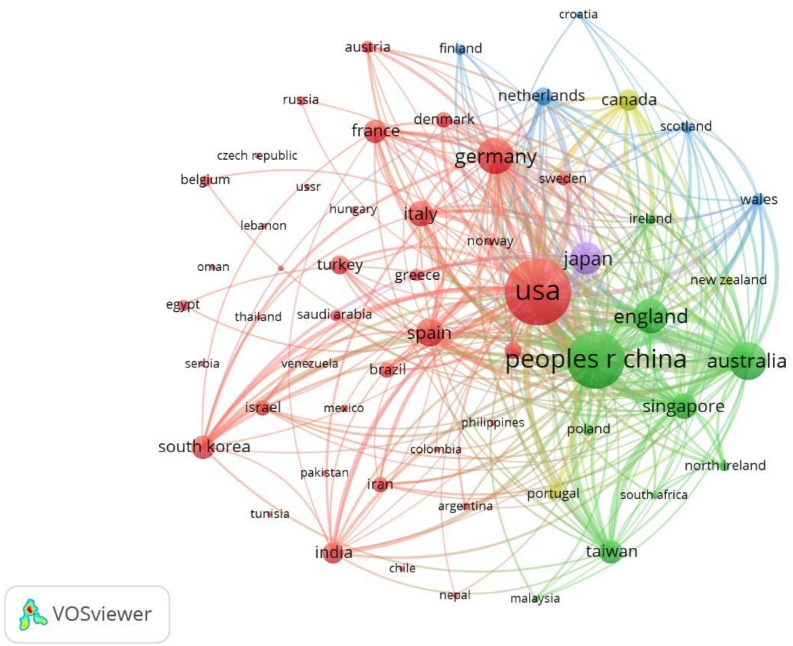

Supplementary Figure 1. Network visualization map of research collaborations between the top active countries. Each country has a contribution of at least 10 documents. There are 57 countries that reached this threshold out of 127 countries that are active in this field.

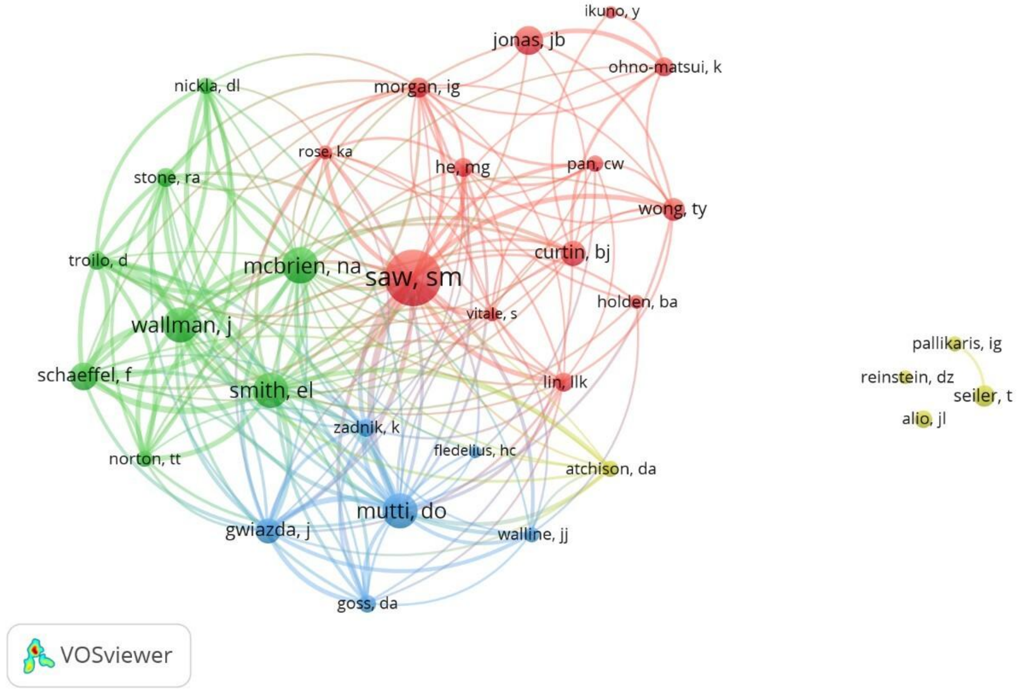

Supplementary Figure 2. Co-authorship network of myopia studies. The minimum number of documents of an author was set as 600. Out of the 71292 authors that were involved in myopia research, 32 authors met the threshold.

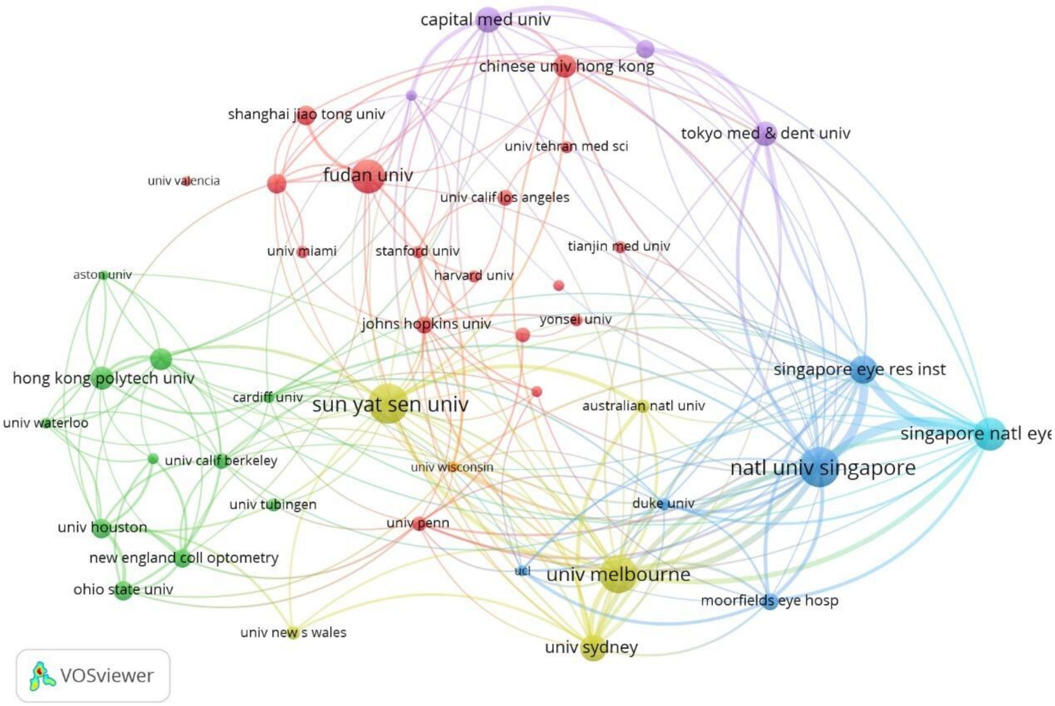

Supplementary Figure 3. Collaboration network of the main research organizations for myopia studies. The minimum number of documents of an organization was set as 65. Out of the 6746 organizations that were involved in myopia research, 45 organizations met the threshold.

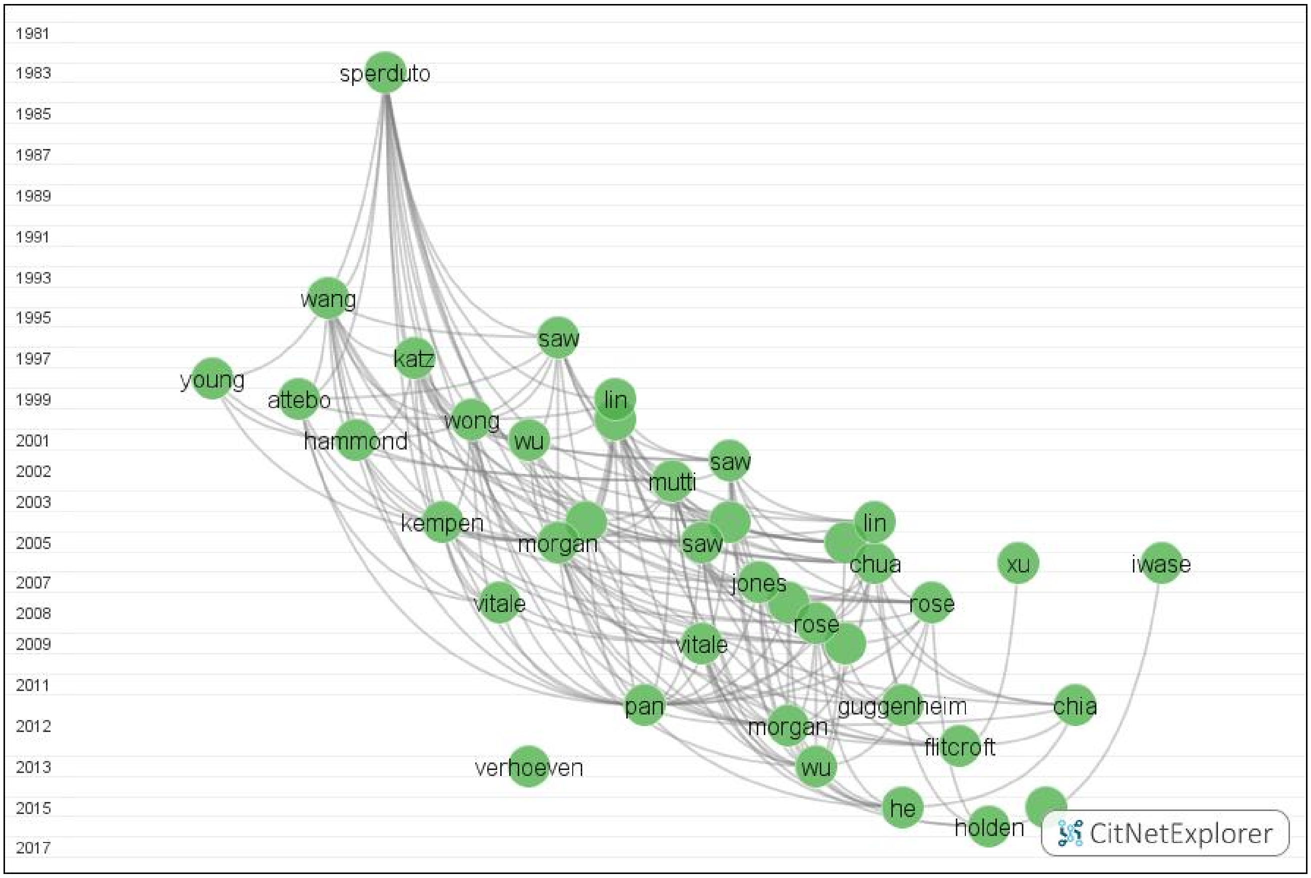

Supplementary Figure 4. Citation network of the prevalence and risk factors of myopia group.

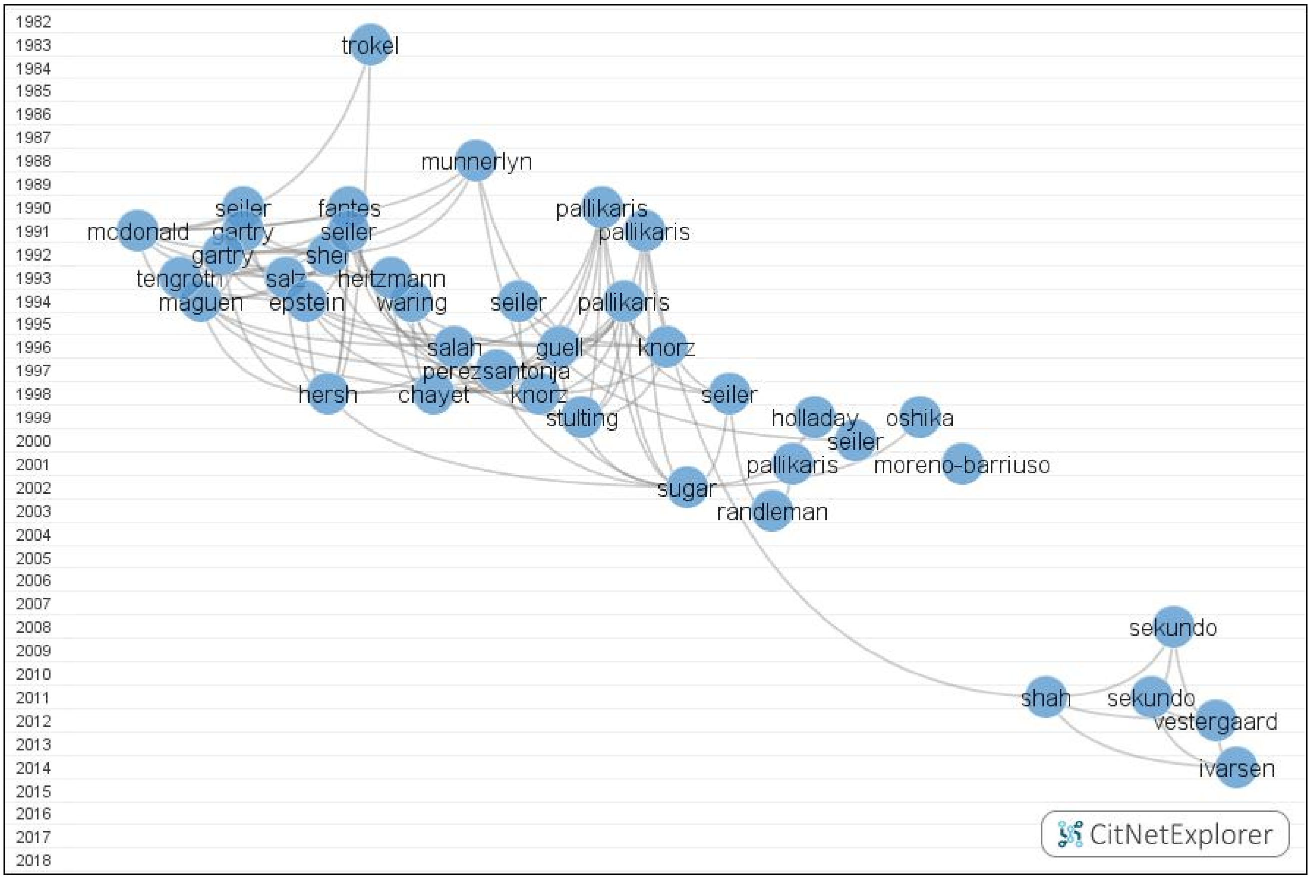

Supplementary Figure 5. Citation network of the surgical control of myopia group.

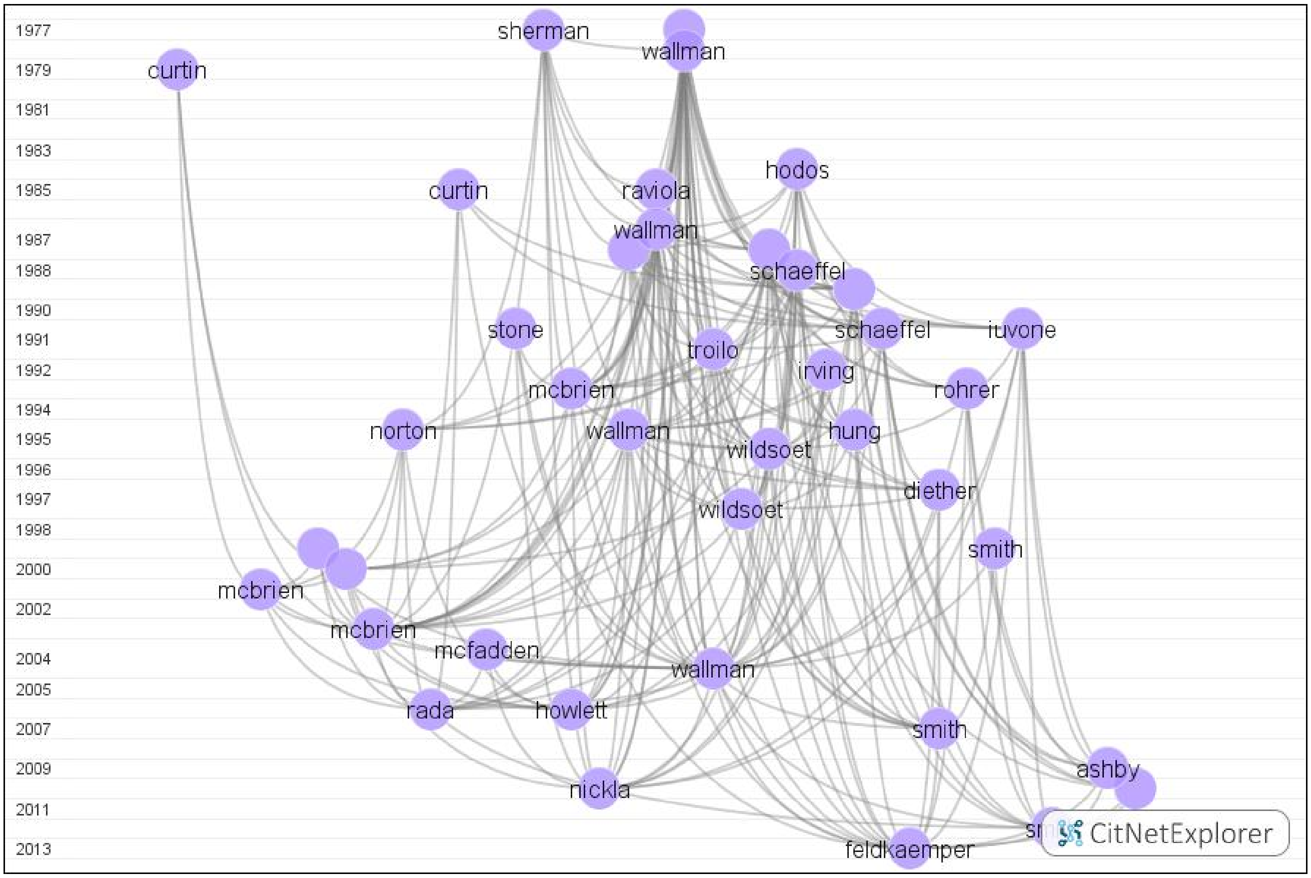

Supplementary Figure 6. Citation network of the pathogenesis of myopia group.

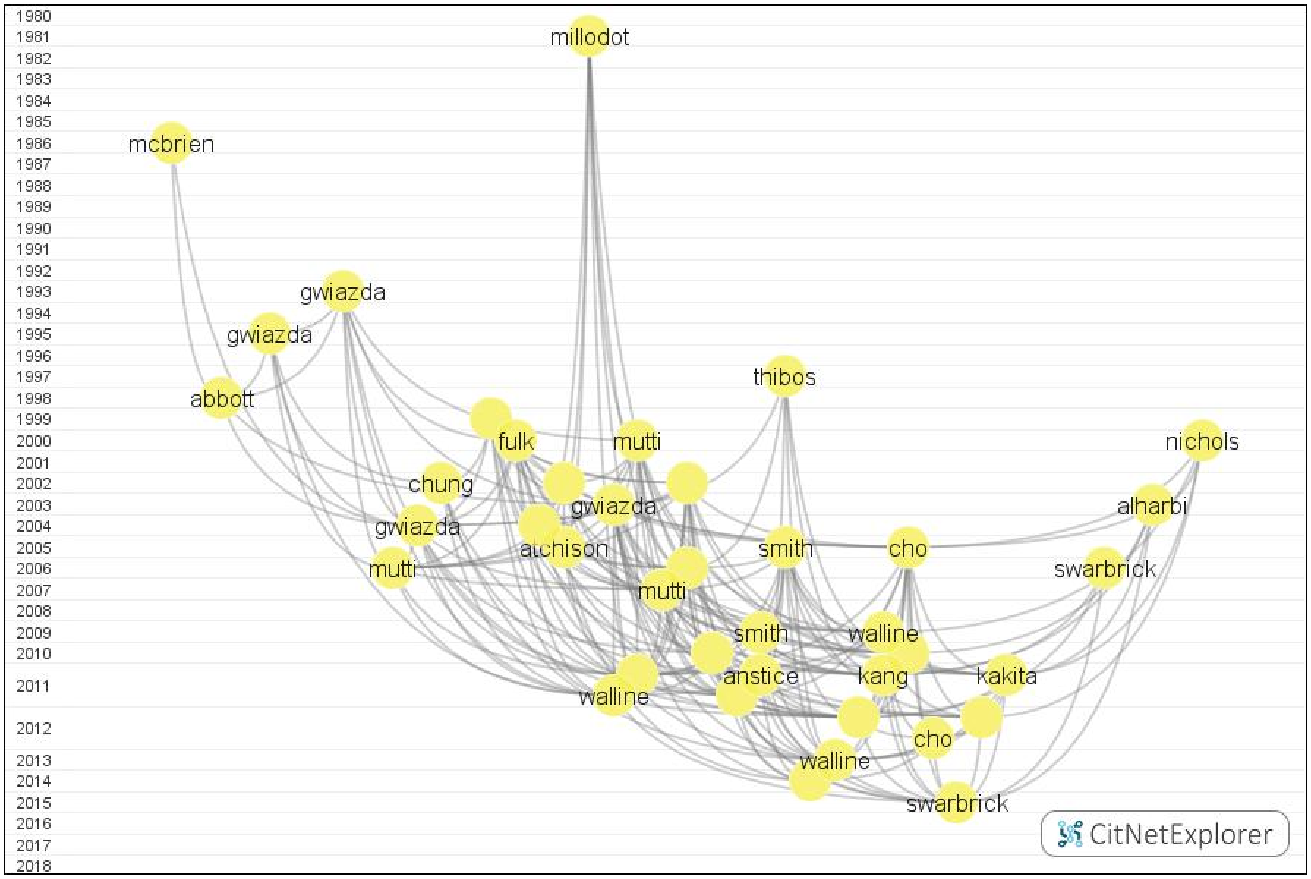

Supplementary Figure 7. Citation network of the optical interventions of myopia group.

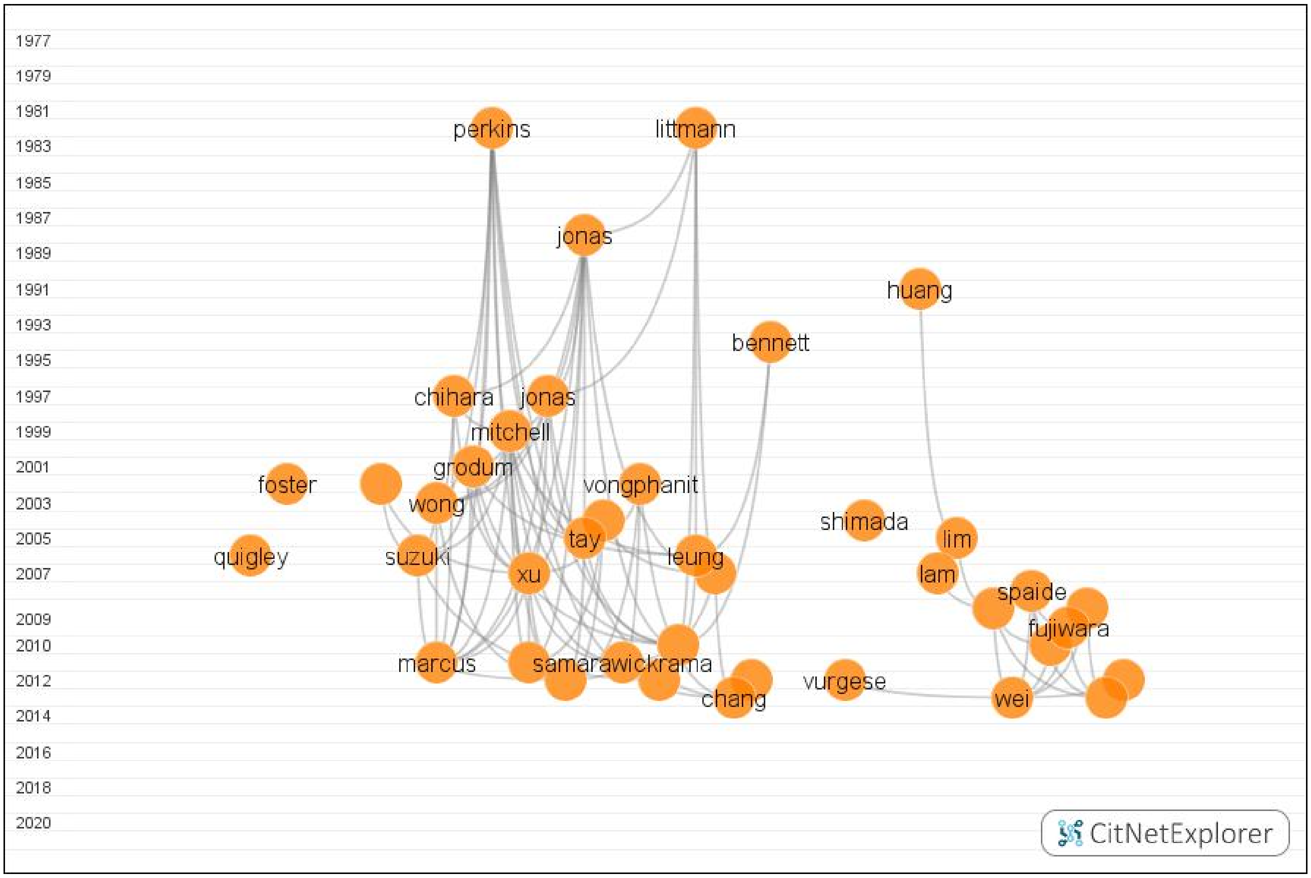

Supplementary Figure 8. Citation network of the myopia and glaucoma group

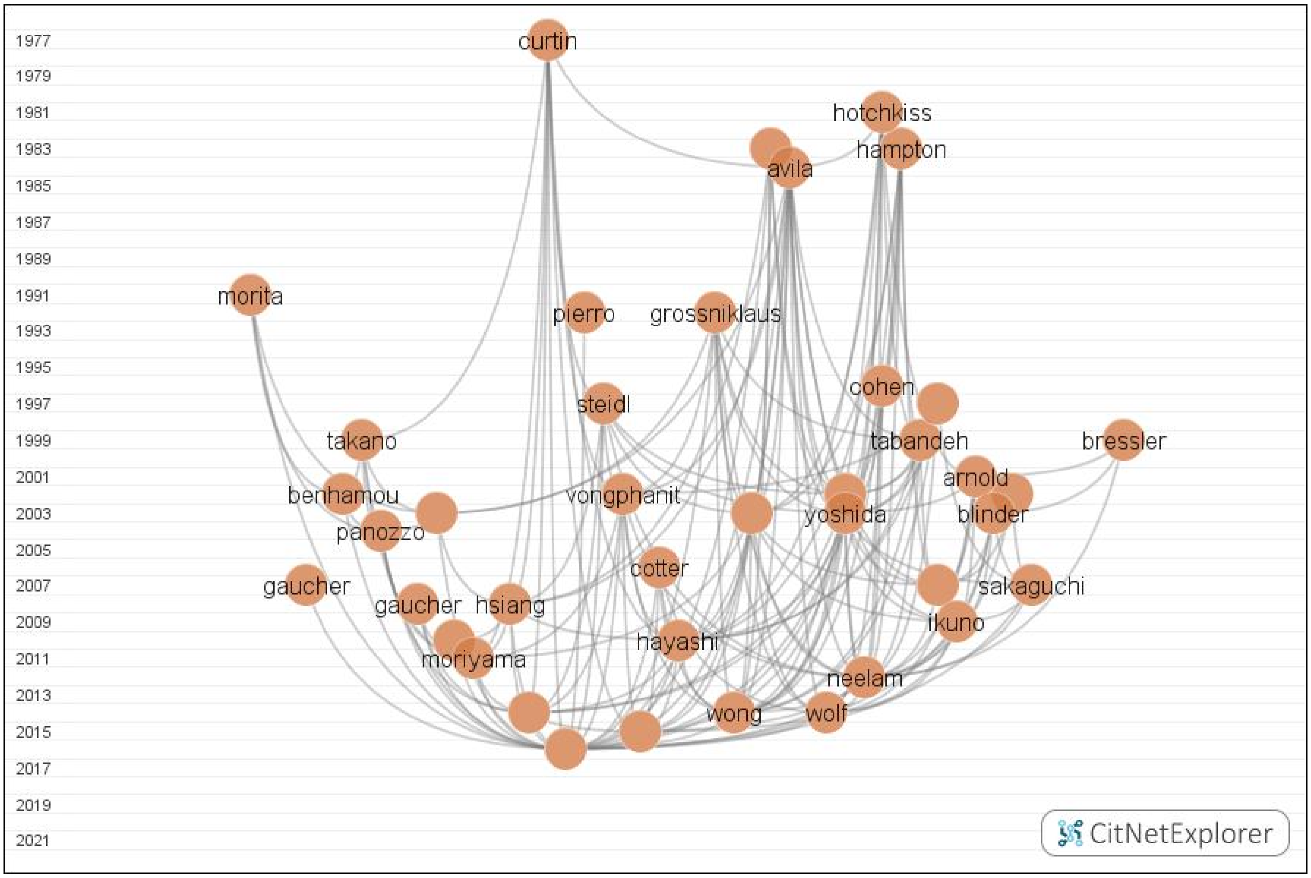

Supplementary Figure 9. Citation network of the pathological myopia group.
